# Intraoperative visualization of lymphatic flow along the ileocolic artery using ICG fluorescence imaging and its implications for right-sided colon cancer surgery

**DOI:** 10.1007/s00464-025-11912-0

**Published:** 2025-07-26

**Authors:** Mamoru Uemura, Chikako Kusunoki, Shoichiro Nakajo, Mao Osaki, Hiroshi Kusafuka, Yuki Sekido, Mitsunobu Takeda, Tsuyoshi Hata, Atsushi Hamabe, Takayuki Ogino, Norikatsu Miyoshi, Yoshinori Kagawa, Mitsuyoshi Tei, Yuichiro Doki, Hidetoshi Eguchi

**Affiliations:** 1 Department of Gastroenterological Surgery, Graduate School of Medicine, The University of Osaka, 2-2 Yamada-Oka, Suita City, Osaka, 565-0871 Japan; 2 Department of Gastroenterological Surgery, Osaka International Cancer Institute, Osaka, Japan; 3 Department of Surgery, Osaka Rosai Hospital, Sakai, Japan

**Keywords:** Right-sided colon cancer, Lymphatic flow, Indocyanine green (ICG) fluorescence imaging, Intraoperative navigation, Lymph node dissection

## Abstract

**Background:**

In colorectal cancer surgery, especially for right-sided colon cancer, understanding the anatomical course of lymphatic flow along the ileocolic artery (ICA) is crucial for safe and oncologically adequate mesenteric dissection. However, the precise spatial relationship between the ICA and the accompanying lymphatic vessels has not been well-established.

**Methods:**

Seventeen patients undergoing laparoscopic right-sided colon cancer surgery were enrolled. Indocyanine green (ICG) was injected into the subserosal layer of the normal ascending colon wall peripheral to the ICA to visualize lymphatic flow. The distance between the lymphatic flow and the ICA was measured intraoperatively using ICG fluorescence imaging.

**Results:**

Lymphatic flow was successfully visualized in 15 of the 17 patients without any complications. After increasing the ICG injection volume to 1.0 mL, lymphatic flow along the ICA was clearly identified in all subsequent 13 cases. The median distance between the lymphatic flow and the ICA was 1.9 cm (interquartile range [IQR], 1.70–2.05 cm). In one case, the flow ran cranially to the ICA and was recorded as 0.0 cm. No major postoperative complications or mesenteric lymph node recurrence were observed during a median follow-up of 3.73 years.

**Conclusions:**

This study measured the distance between the ICA and the lymphatic flow that ran caudal to the artery, and found a distance of up to approximately 2 cm. These findings may serve as a useful anatomical reference for determining a safe and appropriate mesenteric dissection line in right-sided colon cancer surgery.

Surgical treatment plays a central role in the management of colorectal cancer, and appropriate resection margins along with proper lymph node dissection are essential to ensure favorable treatment outcomes. In colon cancer surgery, complete mesocolic excision (CME) with central vascular ligation (CVL) is critical. CME involves the complete resection of the mesocolon, which contains the tumor-associated lymphatic drainage, thereby contributing to improved prognosis through adequate lymphadenectomy [1, 2]. As evidence supporting the importance of CME and CVL, several detailed anatomical studies using cadaveric models have been conducted. These studies have clarified the relationships between vessels and lymphatics, the distribution of lymphatic channels, and the location of central lymph nodes—all of which provide valuable information for determining the appropriate extent of lymphadenectomy and guiding intraoperative decisions in right-sided colon cancer surgery [3–5].

The visualization of lymphatic flow and lymph nodes using indocyanine green (ICG) fluorescence imaging has been reported to improve the understanding of lymphatic pathways and provide valuable guidance for lymphadenectomy in cancer surgery [6].

In cases of right-sided colon cancer requiring lymphadenectomy along the ileocolic artery, the mesentery is dissected parallel to the ileocolic vessels while maintaining an appropriate distance from the vascular pedicle, and the ileocolic vessels are divided at their root to achieve adequate lymph node dissection. However, it remains unclear how far from the ileocolic artery the mesentery should be dissected to ensure oncological safety. Although lymphatic flow in the colon generally proceeds centrally along the supplying arteries [7, 8], the exact distance and course of the lymphatic vessels in relation to these arteries have not been fully elucidated. Determining an oncologically safe mesenteric dissection line based on this anatomical relationship is therefore a key issue in colon cancer surgery.

This study aimed to visualize lymphatic flow along the ICA using ICG fluorescence imaging and to determine an oncologically safe distance from the ICA for mesenteric transection during lymphadenectomy in patients with right-sided colon cancer requiring lymph node dissection in the ICA region.

## Materials and methods

### Patients

Seventeen patients who underwent right hemicolectomy for right-sided colon cancer were included in this study. All patients were confirmed to have no iodine allergy preoperatively. When lymph nodes and lymphatic vessels are infiltrated by cancer cells, lymphatic flow can become obstructed, and the typical fluorescence of ICG may no longer be observed [9]. Therefore, injecting ICG at the tumor site may not allow for accurate visualization of the lymphatic pathway or lymph nodes. To achieve reliable anatomical visualization of lymphatic vessels along the ICA, we considered it essential to inject the tracer (ICG) precisely into the peripheral area of the ICA while confirming anatomical landmarks under direct visualization. A randomized controlled trial in gastric cancer has shown that subserosal injection provides lymph node mapping efficacy comparable to that of submucosal injection, with reduced patient burden [10]. Based on this, we adopted the subserosal injection method under direct vision in this study. Consequently, to accurately identify lymphatic flow along the ICA, ICG was injected into the subserosal layer near the mesenteric attachment site of the intestinal wall at the distal end of the ICA. Only cases without tumors in this region were selected. The clinical characteristics of all 17 patients are listed in Table 1.

**Table 1:** Patient characteristics, surgical procedures, and lymphatic flow findings by ICG injection

Case No	Age (years)	Sex	Tumor locaton	Operative procedures	pTMN Classification	Injected ICG volume (ml)	Max distance between ICA and ICG-highlighted lymphatic vessel (cm)
1	69	F	Ascending	Right hemicolectomy	T4bN1bM1c	0.3	2.5
2	71	M	Cecum	Ileocecal resection	T4aN1aM0	0.3	ND
3	77	F	Transverse	Right hemicolectomy	T4aN1aM1c	0.4	ND
4	73	F	Ascending	Right hemicolectomy	T3N0M0	0.6	1.8
5	83	M	Ascending	Right hemicolectomy	T3N1bM0	1.0	1.6
6	88	F	Cecum	Ileocecal resection	T4aN0M0	1.0	1.8
7	75	M	Ascending	Right hemicolectomy	T4aN0M0	1.0	1.9
8	83	F	Ascending	Right hemicolectomy	T3N0M0	1.0	1.8
9	80	F	Ascending	Right hemicolectomy	T4aN1aM0	1.0	2.4
10	69	M	Ascending	Right hemicolectomy	T1bN0M0	1.0	2.0
11	72	F	Cecum	Ileocecal resection	T4aN2bM1b	1.0	2.5
12	68	M	Ascending	Right hemicolectomy	T3N2bM0	1.0	2.1
13	64	M	Cecum	Ileocecal resection	T3N0M0	1.0	0.0
14	61	F	Ascending	Right hemicolectomy	T1bN0M0	1.0	2.0
15	49	M	Ascending	Right hemicolectomy	T2N1aM0	1.0	1.2
16	66	M	Ascending	Right hemicolectomy	T3N1aM0	1.0	1.0
17	64	F	Transverse	Right hemicolectomy	T4aN2bM0	1.0	2.0

*ICA* ileocecal artery, *ND* not detected

### Injection of ICG and Lymphatic flow observation using near-infrared fluorescence imaging

**Fig. 1:**
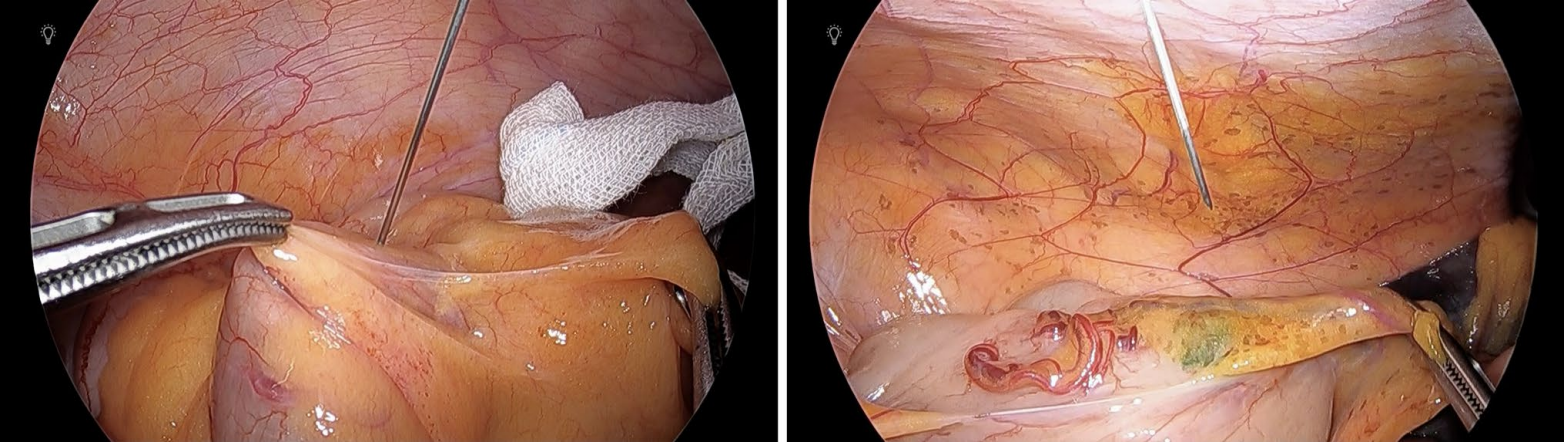
Intraoperative view showing direct injection of ICG into the ascending colon via the abdominal wall

After establishing a pneumoperitoneum at 10 mmHg with CO_2_, and prior to initiating surgical procedures, ICG was injected near the mesenteric attachment of the ascending colon wall at the distal end of the ICA. In all cases, a single injection was performed on the anterior wall of the ascending colon. A stock solution of ICG (Diagnogreen; Daiichi Sankyo, Tokyo, Japan) was prepared by dissolving 25 mg of powdered ICG in 10 mL of sterile water. Using a 23-gauge needle, 1.0 mL of the ICG solution was carefully injected into the intestinal wall through the abdominal wall under laparoscopic guidance (Fig. 1). Subserosal placement was confirmed by the formation of a slight bulge beneath the serosal surface and the visual appearance of green fluorescence through the serosa. In the first four cases, a smaller volume (0.3–0.6 mL) was injected, and lymphatic flow along the ICA was visualized in two of these cases. From the fifth case onward, 1.0 mL was injected, and lymphatic flow was successfully visualized in all cases.

Lymphatic flow observation using near-infrared fluorescence imaging was performed with either the Stryker 1588 or 1688 Advanced Imaging Modalities (AIM) platform (Stryker Endoscopy, Kalamazoo, MI, USA). Both systems consist of an infrared fluorescence (IRF)-enabled LED light source, light cable, camera control unit (CCU), camera head coupler, and a 30° 10 mm laparoscope. These platforms allow real-time visualization of lymphatic flow by superimposing near-infrared (NIR) fluorescence images onto white-light images using an integrated image overlay function. Lymphatic flow appeared as a delayed, fine linear pattern with directional progression, which was clearly distinguishable from venous fluorescence. All fluorescent structures judged to be lymphatic vessels for distance measurement were located within the resected specimens, and postoperative inspection confirmed that they were not veins.

### Surgical procedures

**Fig. 2:**
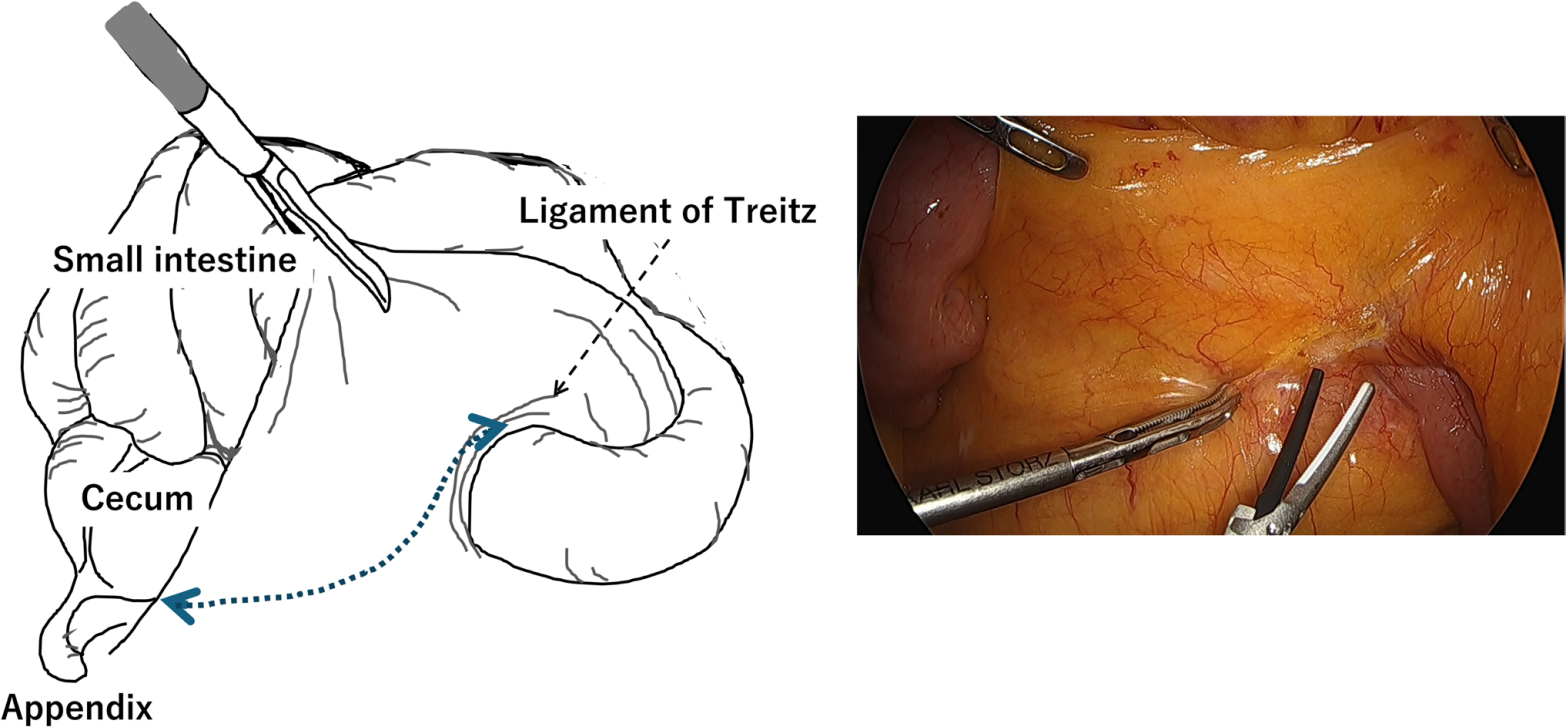
Medial-to-lateral dissection beginning at the retroperitoneal side

**Fig. 3:**
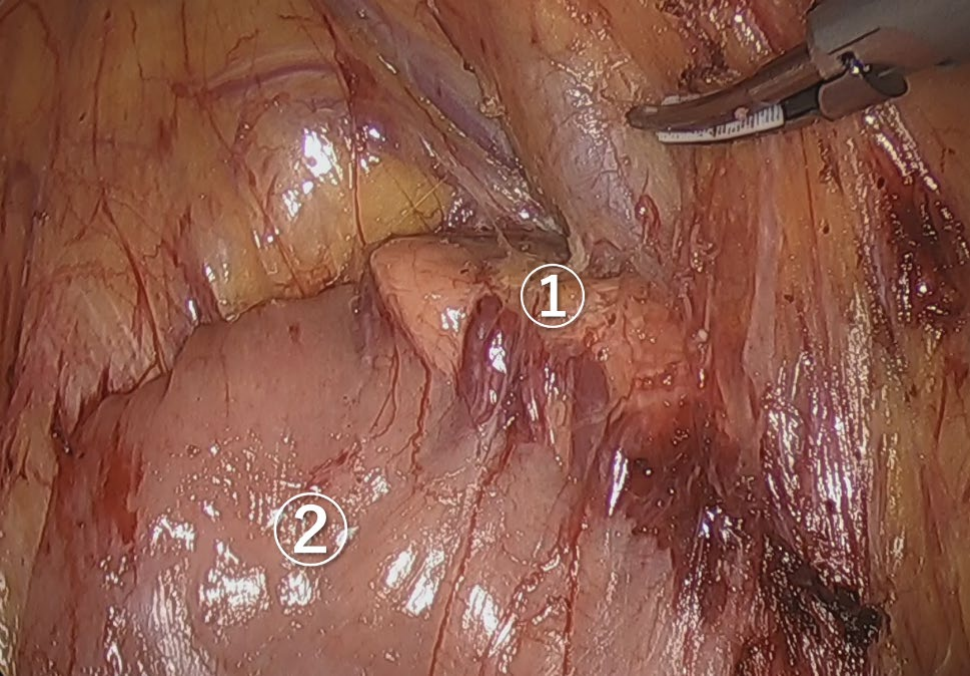
Posterior dissection to dorsally preserve the pancreas and duodenum. ①: Pancreas, ②: Duodenum

The surgical procedure was initiated after the injection of ICG. The patient was placed in the Trendelenburg position with the right side down to shift the small intestine into the upper right quadrant of the abdomen, facilitating exposure of the root of the small bowel mesentery. A surgical field was established to allow a straight and wide view of the mesenteric root (Fig. 2), followed by incision at this site and mobilization of the mesentery from the retroperitoneum. In all cases, this medial-to-lateral approach was employed through the mesenteric root, with posterior dissection along the dorsal mesocolic plane to mobilize the ureter, gonadal vessels, and duodenum dorsally (Fig. 3). This dissection toward the retroperitoneal side was based on a medial-to-lateral approach from the mesenteric root and did not involve intentional identification or dissection of the ureter or gonadal vessels. These structures were passively displaced dorsally during the dissection between the mesentery and retroperitoneal tissues.

**Fig. 4:**
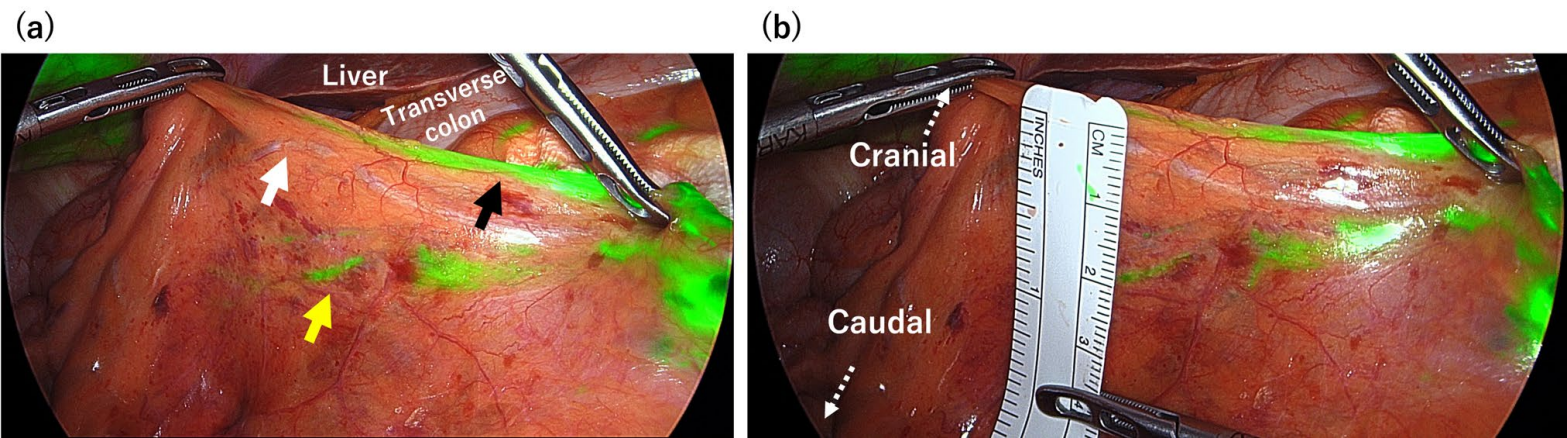
Visualization of lymphatic flow running caudal to the ICA and measurement of the distance between the flow and the ICA. The white arrow indicates the ileocolic artery (ICA). The yellow arrow shows the lymphatic flow running caudally to the ICA, and the black arrow indicates the lymphatic flow running cranially to the ICA

After sufficient dissection from the retroperitoneal side, the ileocolic vascular pedicle was grasped and lifted ventrally to delineate its course. Subsequently, the lymphatic flow along the ileocolic artery was visualized using ICG fluorescence imaging (Fig. 4a), and the maximal distance between the artery and the lymphatic pathway was measured (Fig. 4b).

**Fig. 5:**
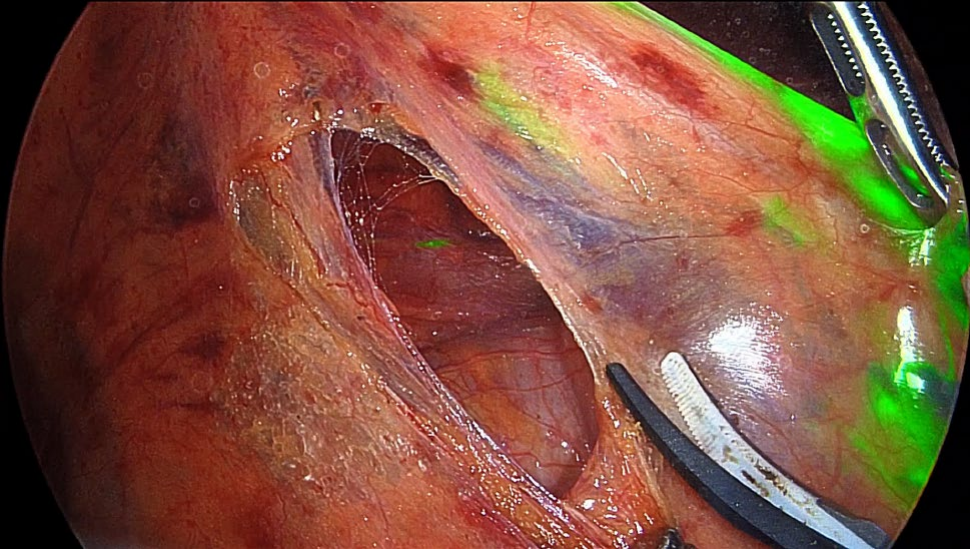
Mesenteric dissection performed along the ICA under visualization of lymphatic flow

To ensure that the identified lymphatic pathway would be included in the resection specimen, the mesentery was incised at a sufficient distance from the vascular pedicle, and a window was created toward the retroperitoneal side (Fig. 5). This window was then extended proximally along the ileocolic artery toward the root of the ileocolic vessels. Upon reaching the left margin of the superior mesenteric vein (SMV), the SMV was clearly exposed, and the root of the ileocolic vessels was divided.

### Ethics approval statement

Approval of the research protocol: This study was approved by the Ethics Committee of Osaka University and conforms to the provisions of the Declaration of Helsinki (16,379–3).

*Informed consent* All informed consent was obtained from the participants.

## Results

A total of 8 males and 9 females were enrolled, with a median age of 71 years (interquartile range [IQR], 66–77 years). Patient characteristics, including tumor location, surgical procedures performed laparoscopically, and pathological TNM classification (pTNM), are summarized in Table 1. Lymphatic flow was successfully visualized in 15 patients without any complications. After increasing the injection volume of ICG to 1.0 mL, lymphatic flow along the ICA was observed in all 13 subsequent cases. This increase was based on empirical observation; the initial volume of 0.3 mL was selected under the assumption that a smaller amount might suffice for targeted visualization of lymphatic flow along the ICA. However, clearer and more consistent imaging was achieved with 1.0 mL, leading us to adopt it as the standardized volume for all subsequent cases.

In this study, ICG was injected into the bowel wall prior to the start of the surgical procedure. After confirming successful injection and assessing for vascular enhancement, mesenteric dissection was initiated from the root of the mesentery, as described in the Surgical Procedures section. After completing the dissection between the retroperitoneal structures and the bowel/bowel mesentery, lymphatic flow was confirmed when the operative field allowed the ICA to be clearly visualized in a straight course. Thus, the timing of lymphatic visualization was not monitored continuously but was determined based on the point at which a sufficient operative field was achieved. The time from ICG injection to observation was a median of 32 min (IQR: 30–44 min). The median distance between the lymphatic flow and the ICA was 1.9 cm (IQR, 1.70–2.05 cm) as shown in Table 1. In Case 13, the visualized lymphatic flow ran cranial rather than caudal to the ICA, and was therefore recorded as 0.0 cm according to the current evaluation method. When the lymphatic flow along the ICA was visualized, the mesenteric dissection line was determined at a distance from the ICA to avoid damaging the visualized lymphatic vessels. In all cases, the postoperative course was uneventful, with no intraoperative complications or postoperative complications classified as Clavien–Dindo grade III or higher.

The median postoperative follow-up period was 3.73 years (IQR, 3.37–4.13 years), and no mesenteric lymph node recurrence was observed in any case. Two patients died during follow-up: Case No. 4 died of an unrelated disease 0.87 years after surgery, and Case No. 11 died of the primary disease 0.57 years after surgery.

## Discussion

In colorectal cancer surgery, the importance of CME and central vascular ligation CVL for oncological radicality has been widely acknowledged [1, 2]. Accordingly, the surgical technique aims to achieve mesenteric dissection based on meticulous anatomical recognition. When resecting the bowel, the mesentery must be transected perpendicular to the bowel axis. This leads to dissection in a direction parallel to the supplying arteries of the bowel and tumor. During this process, surgeons take into consideration the presence of lymph nodes and lymphatic vessels along the arteries and attempt to maintain an appropriate distance from the arteries when dividing the mesentery. However, no clear standard exists regarding the optimal distance from the arteries to ensure inclusion of the perivascular lymphatic structures on the resected side. As a result, the extent of dissection is often determined by the empirical judgment of each surgeon.

In cases of right-sided colon cancer, depending on tumor location, dissection of lymph nodes along the ICA may be required. During ileocecal resection or right hemicolectomy, the mesentery is divided in a direction parallel to the ICA. However, no definitive evidence has clarified how far the lymphatic flow maintains its distance from the artery. To ensure oncological safety in right-sided colon cancer surgery, it is essential to understand the spatial relationship between the lymphatic flow and the supplying artery. Although a similar investigation of lymphatic flow is also necessary for the middle colic artery (MCA) region, anatomical variations in the course of the MCA make standardized evaluation difficult. Therefore, in this study, we focused solely on the lymphatic flow along the ileocolic artery. Transection of the mesentery along the ICA is a common step in surgery for right-sided colon cancer. We consider it clinically important to clarify the pattern of lymphatic flow in this area. Although several reports have described the use of ICG fluorescence imaging for the detection of lymph nodes and the evaluation of lymphadenectomy in colorectal cancer [6, 11, 12], no study has visualized the longitudinal lymphatic flow along the arteries to discuss a safe dissection line in the mesentery.

The ICA is present in all patients, and although its course shows some anatomical variation, safe handling of the mesentery around it is a common requirement in surgery for right-sided colon cancer [13]. Therefore, as shown in this study, the concept of understanding the spatial relationship between the ICA and the parallel lymphatic flow—and dissecting the mesentery in a manner that avoids injuring these lymphatics—is universally important in right-sided colon cancer surgery. The intraoperative identification of lymphatic vessels running caudally along the ICA aligns with the findings from cadaveric studies by Nesgaard et al. [3], indicating a consistent anatomical pattern. These observations contribute to a more comprehensive understanding of the lymphatic anatomy of the right colon. On the other hand, metastasis to lymph nodes in the D3 area has been reported as a potential indicator of systemic disease [14], and the significance and extent of dissection in such cases should be carefully considered. In the central area (D3), postmortem studies have shown that lymph nodes may exist even posterior to the superior mesenteric artery and vein [5], making the determination of the optimal extent of central lymph node dissection both clinically and anatomically important, yet extremely challenging. The lymphatic flow observed in this study ran through what can be considered the intermediate to central mesenteric region. In surgery for right-sided colon cancer, this region is typically resected regardless of tumor location. Therefore, we believe that the findings of this study provide useful reference data for defining the resection area that is commonly necessary in all such cases.

In recent years, with the advancement of camera systems used in laparoscopic and robot-assisted surgery, ICG fluorescence imaging has been increasingly utilized to evaluate not only blood flow but also lymphatic flow and lymph nodes. In particular, there is emerging evidence suggesting that perfusion assessment with ICG is useful in preventing anastomotic leakage [15, 16]. However, there is an important limitation in the use of ICG for lymphatic imaging: lymphatic flow or lymph nodes may not be visualized in certain regions due to lymphatic obstruction caused by the tumor. Therefore, the absence of ICG-based visualization does not justify omission of lymphadenectomy in those areas [9]. With regard to lymphatic flow, rather than aiming to visualize potentially metastatic lymph nodes, it is more meaningful to understand the normal lymphatic anatomy. Applying such anatomical knowledge to cancer surgery appears to be the most practical and clinically useful application of ICG-based lymphatic imaging.

In this study, although the number of cases was limited and definitive conclusions are difficult to draw, the measured distance between the ICA and the corresponding lymphatic flow showed relatively consistent values—except for case No. 13, in which the lymphatic flow ran only cranial to the ICA and the measured distance was 0 cm. The lymphatic flow located on the cranial side of the ICA is typically included in the resected specimen during right-sided colon cancer surgery. Therefore, the precise distance between the ICA and the cranial lymphatic flow is considered to be of limited clinical significance.

As described in the Results section, no mesenteric lymph node recurrence was observed during the follow-up period in any of the patients included in this study. It should be noted that this observation was not intended to validate the clinical usefulness or oncological sufficiency of the resection strategy based on ICG-guided lymphatic flow. Rather, it provides reassurance that the dissection line guided by ICG visualization did not result in oncologically adverse outcomes in this cohort.

Since ICG was not directly injected into the tumor, the lymphatic flow observed in this study does not represent tumor-specific drainage. Rather, the ICG was injected into the subserosal layer of the normal ascending colon wall distal to the ICA, allowing visualization of the natural lymphatic flow pattern along the ICA. This approach was selected to better understand the normal lymphatic anatomy. To our knowledge, no previous report has provided data on the distance between the ICA and its associated lymphatic flow. Therefore, despite the limitations, the current findings may offer a valuable reference for planning oncologically appropriate dissection in right-sided colon cancer surgery. This study was not intended to clarify how the measured distance from the ICA relates to oncological safety, such as lymph node metastasis or local recurrence. Instead, the distance is proposed as a potential reference during surgery. Further prospective studies are needed to verify whether this distance can serve as an oncologically safe margin.

In conclusion, this study measured the distance between the ICA and the lymphatic flow running on its caudal side, which was found to be up to approximately 2 cm. These findings provide a useful anatomical reference that may help surgeons determine a safe and appropriate mesenteric dissection line during right-sided colon cancer surgery.

## References

[1] West NP, Hohenberger W, Weber K, Perrakis A, Finan PJ, Quirke P (2010) Complete mesocolic excision with central vascular ligation produces an oncologically superior specimen compared with standard surgery for carcinoma of the colon. J Clin Oncol 28(2):272–278 19949013 10.1200/JCO.2009.24.1448

[2] West NP, Kobayashi H, Takahashi K, Perrakis A, Weber K, Hohenberger W et al (2012) Understanding optimal colonic cancer surgery: comparison of Japanese D3 resection and European complete mesocolic excision with central vascular ligation. J Clin Oncol 30(15):1763–1769 22473170 10.1200/JCO.2011.38.3992

[3] Nesgaard JM, Stimec BV, Soulie P, Edwin B, Bakka A, Ignjatovic D (2018) Defining minimal clearances for adequate lymphatic resection relevant to right colectomy for cancer: a post-mortem study. Surg Endosc 32(9):3806–3812 29435757 10.1007/s00464-018-6106-3

[4] Vasic T, Stimec MB, Stimec BV, Kjaestad E, Ignjatovic D (2025) Jejunal lymphatic and vascular anatomy defines surgical principles for treatment of Jejunal tumors. Dis Colon Rectum 68(5):553–561 39936801 10.1097/DCR.0000000000003644 PMC11999094

[5] Spasojevic M, Stimec BV, Dyrbekk AP, Tepavcevic Z, Edwin B, Bakka A et al (2013) Lymph node distribution in the d3 area of the right mesocolon: implications for an anatomically correct cancer resection. A postmortem study. Dis Colon Rectum 56(12):1381–1387 24201392 10.1097/01.dcr.0000436279.18577.d3

[6] Watanabe J, Ota M, Suwa Y, Ishibe A, Masui H, Nagahori K (2017) Evaluation of lymph flow patterns in splenic flexural colon cancers using laparoscopic real-time indocyanine green fluorescence imaging. Int J Colorectal Dis 32(2):201–207 27695977 10.1007/s00384-016-2669-4

[7] Kim JY (2018) The lymphatic spread of colon cancer. In: Kim N, Sugihara K, Liang JT (eds) Surgical treatment of colorectal cancer. Springer, Singapore, pp 241–9.

[8] Bertelsen CA, Miskovic D (2021) Surgical anatomy of the colon and complete mesocolic excision. In: Baatrup G (ed) Multidisciplinary treatment of colorectal cancer. Springer, Cham, pp 141–55.

[9] Sato Y, Satoyoshi T, Okita K, Kyuno D, Hamabe A, Okuya K et al (2021) Snapshots of lymphatic pathways in colorectal cancer surgery using near-infrared fluorescence, in vivo and ex vivo. Eur J Surg Oncol 47(12):3130–3136 34373159 10.1016/j.ejso.2021.07.025

[10] Chen QY, Zhong Q, Li P, Xie JW, Liu ZY, Huang XB et al (2021) Comparison of submucosal and subserosal approaches toward optimized indocyanine green tracer-guided laparoscopic lymphadenectomy for patients with gastric cancer (FUGES-019): a randomized controlled trial. BMC Med 19(1):276 34702260 10.1186/s12916-021-02125-y PMC8549272

[11] Nishigori N, Koyama F, Nakagawa T, Nakamura S, Ueda T, Inoue T et al (2016) Visualization of lymph/blood flow in laparoscopic colorectal cancer surgery by ICG fluorescence imaging (Lap-IGFI). Ann Surg Oncol 23(Suppl 2):S266–S274 25801355 10.1245/s10434-015-4509-0

[12] Ueda K, Ushijima H, Kawamura J (2023) Lymphatic flow mapping during colon cancer surgery using indocyanine green fluorescence imaging. Minim Invasive Ther Allied Technol 32(5):233–239 36628437 10.1080/13645706.2022.2164468

[13] Nesgaard JM, Stimec BV, Bakka AO, Edwin B, Ignjatovic D, Group RCCs (2015) Navigating the mesentery: a comparative pre- and per-operative visualization of the vascular anatomy. Colorectal Dis 17(9):810–8 25988347 10.1111/codi.13003

[14] Banipal GS, Stimec BV, Andersen SN, Edwin B, Nesgaard JM, Saltyte Benth J et al (2024) Are metastatic central lymph nodes (D3 volume) in right-sided colon cancer a sign of systemic disease? A sub-group analysis of an ongoing multicenter trial. Ann Surg 279(4):648–656 37753647 10.1097/SLA.0000000000006099 PMC10922660

[15] Watanabe J, Takemasa I, Kotake M, Noura S, Kimura K, Suwa H et al (2023) Blood perfusion assessment by indocyanine green fluorescence imaging for minimally invasive rectal cancer surgery (EssentiAL trial): a randomized clinical trial. Ann Surg 278(4):e688–e694 37218517 10.1097/SLA.0000000000005907 PMC10481925

[16] Lucarini A, Guida AM, Orville M, Panis Y (2024) Indocyanine green fluorescence angiography could reduce the risk of anastomotic leakage in rectal cancer surgery: a systematic review and meta-analysis of randomized controlled trials. Colorectal Dis 26(3):408–416 38247221 10.1111/codi.16868

